# Serum metabolite profile associates with the development of metabolic co-morbidities in first-episode psychosis

**DOI:** 10.1038/tp.2016.222

**Published:** 2016-11-15

**Authors:** T Suvitaival, O Mantere, T Kieseppä, I Mattila, P Pöhö, T Hyötyläinen, J Suvisaari, M Orešič

**Affiliations:** 1 Steno Diabetes Center, Gentofte, Denmark; 2 Mental Health Unit, National Institute for Health and Welfare, Helsinki, Finland; 3 Department of Psychiatry, McGill University, Montréal, QC, Canada; 4 Bipolar Disorders Clinic, Douglas Mental Health University Institute, Montréal, QC, Canada; 5 Department of Psychiatry, Helsinki University and Helsinki University Hospital, Helsinki, Finland; 6 Faculty of Pharmacy, University of Helsinki, Helsinki, Finland; 7 Department of Chemistry, Örebro University, Örebro, Sweden; 8 Turku Centre for Biotechnology, University of Turku and Åbo Akademi University, Turku, Finland

## Abstract

Psychotic patients are at high risk for developing obesity, metabolic syndrome and type 2 diabetes. These metabolic co-morbidities are hypothesized to be related to both treatment side effects as well as to metabolic changes occurring during the psychosis. Earlier metabolomics studies have shown that blood metabolite levels are predictive of insulin resistance and type 2 diabetes in the general population as well as sensitive to the effects of antipsychotics. In this study, we aimed to identify the metabolite profiles predicting future weight gain and other metabolic abnormalities in psychotic patients. We applied comprehensive metabolomics to investigate serum metabolite profiles in a prospective study setting in 36 first-episode psychosis patients during the first year of the antipsychotic treatment and 19 controls. While corroborating several earlier findings when comparing cases and controls and the effects of the antipsychotic medication, we also found that prospective weight gain in psychotic patients was associated with increased levels of triacylglycerols with low carbon number and double-bond count at baseline, that is, lipids known to be associated with increased liver fat. Our study suggests that metabolite profiles may be used to identify the psychotic patients most vulnerable to develop metabolic co-morbidities, and may point to a pharmacological approach to counteract the antipsychotic-induced weight gain.

## Introduction

Patients with first-episode psychosis (FEP) have high risk of weight gain, glucose dysregulation and dyslipidemias during the early stages of illness.^1, 2^ Although some of these cardiometabolic risk factors may be present already before the initiation of treatment,^3^ cardiovascular risk increases after exposure to antipsychotic drugs.^4^ Weight gain is most rapid during the first months of treatment, after which weight gradually stabilizes.^5^ Antipsychotic drugs differ in their propensity to cause weight gain and other metabolic side effects.^4, 6^ Unfortunately, clozapine and olanzapine, which are among the most efficacious antipsychotics, are also the most likely to cause cardiometabolic side effects.^4, 6, 7, 8^ However, there is significant individual variation in the propensity to antipsychotic-induced weight gain and other metabolic alterations, some of which may be genetically mediated.^9, 10, 11^ To optimize the effectiveness and tolerability of treatment, biomarkers for early identification of psychotic patients at high risk of weight gain and the development of associated cardiometabolic co-morbidities would be highly useful in clinical practice.

Metabolomics, and lipidomics as one of its branches, are promising new tools for the identification of biomarkers for both etiopathology of psychotic disorders and antipsychotic side effects.^12, 13, 14, 15, 16, 17^ Metabolomics has also had an important role to unravel putative biomarkers and underlying pathways in several other diseases of the central nervous system,^18^ including major depressive disorder^19, 20^ as well as Alzheimer's^21, 22, 23, 24^ and Parkinsons^25, 26, 27^ diseases.

In the general population, specific metabolites are also predictive of development of insulin resistance,^28^ non-alcoholic fatty liver disease^29^ and type 2 diabetes.^30, 31^ All these conditions are known co-morbidities of psychotic disorders. However, previous studies have not investigated metabolomic profiles which might be valuable to identify the risk of early weight gain or other metabolic abnormalities in psychotic patients. Here we set out to investigate aberrations in blood metabolite levels in FEP patients at early stages of the antipsychotic treatment, and followed-up the development of the patients in order to associate the observed aberrations with clinical outcomes.

## Materials and Methods

### Ethics statement

The study protocol was approved by the Ethics Committee of the Hospital District of Helsinki and Uusimaa (257/12/03/03/2009) and by the institutional review boards of the National Institute for Health and Welfare, Helsinki, Finland, and the University of Helsinki, and all participants gave a written informed consent. Patient's capacity to give informed consent was assessed by the treating psychiatrist.

### Clinical study protocol

The study sample and protocol have been previously described in detail in a previous publication.^32^ Patients (aged 18 to 40 years) with FEP were recruited from the catchment area of the Helsinki University Hospital. Psychosis was defined as receiving a score of at least 4 in the items assessing delusions or hallucinations in the Brief Psychiatric Rating Scale-Extended (BPRS-E).^33^ Patients were assessed clinically three times. The baseline assessment was done as soon as the patient had entered treatment and was able to give informed consent, and the follow-ups were done at 2 and 12 months. Each assessment included BPRS-E. In addition, diagnostic assessment was done at 2 months and 1-year follow-up based on the Research Version of the Structured Clinical Interview for DSM-IV and all information from medical records, and DSM-IV diagnosis was done by a senior psychiatrist (JS) together with the interviewer. Data were also gathered on sociodemographic factors, medication, substance use, physical activity, diet and smoking, and the interviewer measured weight, height, blood pressure and waist circumference. Global Assessment of Functioning (GAF) was used as an overall measure of psychological, social and occupational functioning.^32^

Controls were recruited from the Population Register Centre. Their baseline assessment was identical to the patients' protocol.

#### Blood samples

A fasting blood sample (serum and plasma) was collected on the morning following the interview at 8 to 10 am at baseline, 2 months, and 1 year, for patients and at baseline for controls. Samples were immediately aliquoted and stored at −80 ^o^C.

Serum total cholesterol, high-density lipoprotein cholesterol, triglycerides, apolipoprotein A-I (ApoA-I) and B (ApoB) and plasma glucose were measured with enzymatic assays by the Abbott Architect ci8200 analyzer (Abbott Laboratories, Abbott Park, IL, USA). Apolipoprotein A-I (ApoA-I) and B were determined with immunoturbimetric assays (Abbott) and high sensitivity C-reactive protein (hs-CRP) with latex turbidometric immunoassay (Sentinel, Milan, Italy). Insulin and C-peptide were measured with chemiluminescent microparticle immunoassays (Abbott). Low-density lipoprotein cholesterol was calculated by the Friedewald formula. The mean inter-assay coefficient of variations (CVs) for cholesterol, high-density lipoprotein cholesterol, triglycerides, and glucose were 1.0, 2.2, 1.5, and 1.4%. The mean CVs for ApoA-I, ApoB, hs-CRP, insulin, and C-peptide were 1.8, 2.0, 4.3, 2.4, and 2.5%, respectively.^32^

### Analysis of polar metabolites

Serum polar metabolites were analyzed using comprehensive two-dimensional gas chromatography combined with time-of-flight mass spectrometry (GC × GC–TOFMS, a LECO Pegasus 4D from Leco, St. Joseph, MI, USA) with a method described in an earlier publication.^34^ In short, protein precipitation by methanol was followed by two step derivatization using methoximation and silylation. A set of internal standards (C17:0, valine-d8, succinic acid-d4, n-alkane mixture and 4,4'-bibromooctafluorobiphenyl) were added to the serum samples.

ChromaTOF vendor software (LECO) was used for within-sample data processing, and Guineu software was used for alignment, normalization and peak matching across samples.^34^ The normalization was performed by correction for internal standards, and specific target metabolites were additionally quantified using external calibration curves. Other mass spectra from the GC × GC–TOFMS analysis were searched against the NIST Mass Spectral Library and the Golm database, using also retention index data in the identification. Control serum samples were analyzed together with the case samples.

All serum metabolite peaks that were present (had a non-zero value) in more than 50% of the samples, including the unidentified ones, were included in the data analyses. We reasoned that inclusion of complete data as obtained from the platform best represent the global metabolome covered by the platform. The unidentified peaks were annotated with their structural class based on their mass spectra.^34^

### Analysis of molecular lipids

Serum molecular lipids were analyzed using ultra performance liquid chromatography coupled with time-of-flight mass spectrometry (UPLC-QTOFMS Q-Tof Premier mass spectrometer, Waters, Milford, MA, USA) with a methodology described earlier.^35^ The samples were extracted with a chloroform-methanol mixture after the addition of internal standards containing LPC(17:0), PC(17:0/17:0), PE(17:0/17:0), Cer(d18:1/17:0) (Avanti Polar Lipids, Alabaster, AL, USA), and TG(17:0/17:0/17:0) (Larodan Fine Chemicals, Malmö, Sweden). External standard mixture containing (LPC(16:0D_3_), PC(16:0/16:0D_6_) and TG(16:0/16:0/16:0-^13^C3) (Larodan Fine Chemicals, Malmö, Sweden) was added after the extraction. The data processing using MZmine 2 [ref. 36] included the alignment of peaks, peak integration, normalization, and peak identification. Lipids were identified using an internal spectral library or with tandem MS., The data were normalized using lipid class-specific internal standards, as described in a previous publication.^35^ Sphingomyelins were normalized with the PC standard. Again, all lipidomic peaks, including the unidentified ones, were included in the data analyses. Control serum samples and extracted standard samples were analyzed together with the study samples.

### Statistical analysis

Statistical analyses were done using the R statistical language^37^ (version 3.2.3). All statistical analyses of the lipidomic and metabolomic data were based on log_2_-transformed intensity data. For improved global interpretability and for the purpose of multivariate analysis, the transformed data were centered and scaled based on the variable-specific mean and standard deviation of the control samples.

The lipidomic and metabolomic variables were clustered using the infinite Gaussian mixture model^38^ with the variational Bayesian inference algorithm^39^ implemented in the netresponse^40^ R package. The clusters were inferred from the data of the control samples. The hyper-parameters of the model were optimized based on the model fit, which was quantified by the lower bound of the data likelihood. For further analysis, the best-fitting model was selected from the 1000 candidate models that were fit with the optimized hyper-parameters, again based on the lower bound of the data likelihood.

The inferred clusters were inspected for the enrichment of compound groups with the binomial test for each cluster-compound group pair at a false discovery rate (FDR)^41^ of 0.01. For the analysis of cluster levels, the sample-specific median over the variables assigned to each cluster was computed. Lipid clusters enriched with triacylglycerides were further analyzed for the enrichment in the number of carbon atoms and in the number of double bonds with the multinomial test at an FDR of 0.01. Cluster-specific medians for the carbon number and the double-bond count were also calculated.

Reported differences between the groups are median Glass' Δ effects on the log-transformed data. Case-control differences were computed between the case group at the three time points (baseline, 2 months and 1 year) and the control group.

Strength of the differences was assessed with a combination of the Mann–Whitney U-test and the bootstrap test for non-zero effect. At each test, 1000 bootstrap samples were drawn and the boundary of a strong difference was set to *P*<0.05 simultaneously for both the tests. Non-paired tests were used for the comparisons between groups of subjects (case vs control, differences between the three treatment groups) and paired tests were used for studying the temporal development of the case subjects over the follow-up.

Metabolite levels at baseline were screened for associations to follow-up changes in the clinical variables: Spearman correlation was computed between cluster levels at baseline and absolute changes in clinical variables between the baseline and the two follow-up time points. Strength of the association was assessed with a combination of the Spearman's exact rank correlation test and the bootstrap test for Spearman correlation coefficient. At each test, 1000 bootstrap samples were drawn and the boundary of a strong association was set to *P*<0.05 simultaneously for both the tests.

The levels of triacylglycerols (TGs) were analyzed for associations to short-term follow-up relative changes in body mass. The association was quantified by computing the Spearman correlation between the baseline level of each compound and the change in the body mass between the baseline and 2-month follow-up time points. These associations were analyzed for the dependence on the two properties of the TG molecules: the carbon number and the double-bond count. Two linear regression models with each of the two feature variables as an independent variable and the association to change in the body mass as a dependent variable were fit. The 95% confidence intervals for the model fit were computed over 1000 bootstrap resamples.

## Results

### Metabolic changes in first-episode psychosis patients

**Figure 1:**
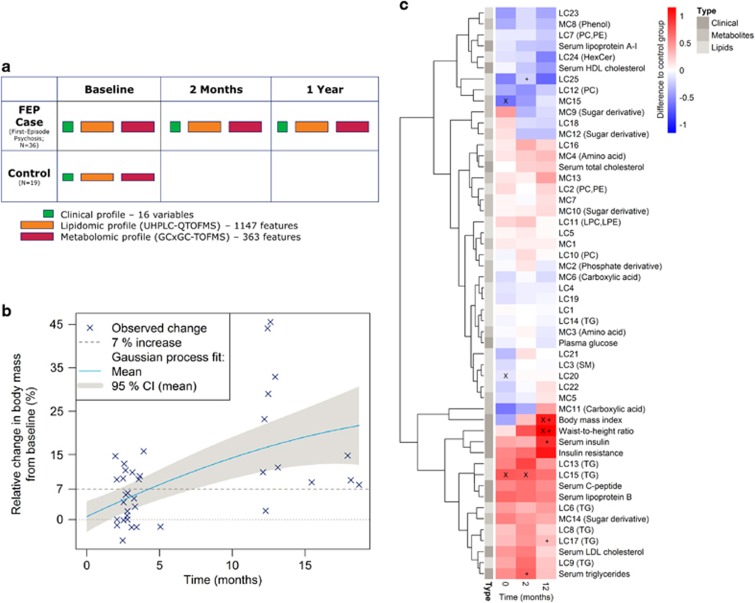
(**a**) Schematic representation of the experimental design. (**b**) Relative weight gain (blue crosses) from baseline as a function of time in the FEP case group. The median increase in body mass was 3 and 11 kg from baseline to the 2-month and 1-year follow-up points, respectively. Nonlinear Gaussian process regression model was fit on the weight gain data to visually highlight the trend. (**c**) Differences in the medians between the case group in the three time points (columns) and control group for clinical variables, metabolite clusters (MCs) and lipid clusters (LCs). Major differences between the case and control groups are highlighted with a diagonal cross ( × ) and major differences between the follow-up time points and the baseline time point among the case group are highlighted with a straight cross (+). Both the tests are based on a coupled Mann–Whitney *U*-test and a bootstrap test of difference with simultaneously *P*<0.05 in both the tests used as a threshold. Statistically significant enrichment (at FDR 0.01) of a functional group in a cluster is shown in parenthesis following the cluster name. FDR, false discovery rate; FEP, first-episode psychosis.

The FEP patients (*n*=36) were examined at baseline as well as at the 2-month and 1-year follow-up time points, whereas the control subjects (*n*=19) were examined only at baseline (Figure 1a). Although not different from the control group at baseline (Table 1; Supplementary Text 1), the patients had worsening metabolic characteristics over time, showing an increase in the body mass, waist circumference and serum insulin during the follow-up (Table 1, Figure 1b). Two metabolomics platforms with broad analytical coverage were applied to all available samples from the FEP patients and controls: (a) global lipidomics platform, based on ultrahigh performance liquid chromatography coupled to mass spectrometry (UHPLC-QTOFMS), covering molecular lipids including phospholipids, sphingolipids and neutral lipids; (b) platform for global profiling of polar metabolites, based on comprehensive two-dimensional gas chromatography coupled to time-of-flight mass spectrometry (GC × GC–TOFMS), covering small molecules such as amino acids, ketoacids, free fatty acids, various other organic acids, sterols and sugars. The final data set included 1148 molecular lipids and 363 polar metabolites from 19 controls and 36 FEP patients (36 subjects at baseline, 24 at 2 months and 12 at 12 months).

In order to reduce the dimensionality of the metabolomic data and to increase its interpretability, we first applied model-based clustering. The clustering algorithm identified 25 lipidomic and 15 polar metabolite clusters (Table 2; LCs and MCs for lipid clusters and metabolite clusters, respectively). Among the clusters, 14 lipid clusters and 10 polar metabolite clusters were enriched with respect to a specific structural group of metabolites. All seven TG-enriched clusters were also enriched with respect to the carbon number and the double-bond count (Table 2), indicating that the TG clusters represent structurally distinct subgroups.

The levels of TGs were generally higher among the FEP patients as compared to controls, both at baseline as well as throughout the follow-up (Figure 1c). The between-group difference was most consistent for a cluster containing polyunsaturated TGs (LC15 with a median of eight unsaturated carbon–carbon bonds) at the baseline and the increase remained strong at the 2-month follow-up. Another TG cluster (LC17 with a median of three unsaturated carbon–carbon bonds) had a consistent increase from the baseline at the 1-year follow-up time point. In contrast, the case group had lower levels of PCs with the most marked decrease at the 2-month follow-up time point in LC12. Also, a metabolite cluster with three unsaturated free fatty acids (MC15 with linolenic, eicosenoic and pentadecanoic acids) was downregulated in cases as compared with controls throughout the follow-up, with the most marked difference already at the baseline time point.

### The effect of antipsychotic medication on metabolomic profiles

The median duration of antipsychotic medication was 27 days at baseline (Supplementary Table 1). The strongest associations between the duration of antipsychotic medication at baseline and the metabolomic profiles at baseline were to the TG clusters LC6 and LC15 (Spearman *r*=0.36 and 0.35, respectively, and both with *P*=0.04 in Spearman exact test but only LC15 with a non-zero 95% Spearman *r* bootsrap confidence interval; Supplementary Tables 2 and 3).

In the follow-up time points, the lipidomic levels were mainly increased in patients administered olanzapine, when compared with other treatments (Supplementary Figure 1). Particularly increased were the two PC clusters (LC7 and LC12). Notably, LC12 was also downregulated in patients at baseline. The lipidomic levels were broadly decreased in patients administered risperidone, when compared with other treatments (Supplementary Figure 2). The decrease was strongest in three bi- and monounsaturated TG clusters (LCs 8, 9 and 13).

### Associations between metabolomic levels and clinical outcomes

**Figure 2:**
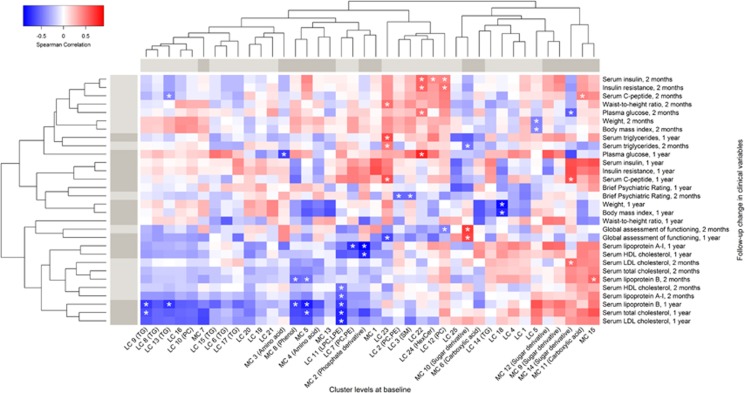
Associations between the baseline levels of the metabolite clusters (columns) and follow-up changes in the clinical variables (rows) among case subjects are shown. Strongest associations (*P*<0.05 with both Spearman exact test and bootstrap test for Spearman correlation) are highlighted with an asterisk. Lipidomic and metabolomic clusters (LCs and MCs, respectively) are annotated with light and dark gray colors in the top margin and so are the 2-month and 1 year clinical change variables in the left margin. Statistically significant enrichment (at FDR 0.01) of a functional group in a cluster is shown in parenthesis following the cluster name. FDR, false discovery rate.

Baseline levels of the PC cluster LC12 were associated to 2-month changes in insulin resistance, serum C-peptide and waist-to-height ratio and inversely associated to changes in the global assessment of functioning (Figure 2). The association to the change in the waist-to-height ratio remained strong in the subjects who were lean at baseline (Spearman correlation *r*=0.69; *P*=0.02).

Two lipidomic clusters with an enrichment of PCs and sphingomyelins (LC2 and LC3, respectively) were inversely associated with a 2-month change in the BPRS (Figure 2). Levels of clusters with mono- and bi-unsaturated TGs (LC9 and LC13), PCs (LC7) and lysophosphatidylcholines (LPCs; LC11) were inversely associated with follow-up changes in cholesterol and lipoproteins. The clusters LC9, 13 and 11 were also inversely associated with the 2-month follow-up changes in insulin among subjects who were lean at baseline (Spearman correlation *r*=−0.59, *P*=0.05; *r*=−0.68, *P*=0.02; and *r*=−0.67, *P*=0.03, respectively).

**Figure 3:**
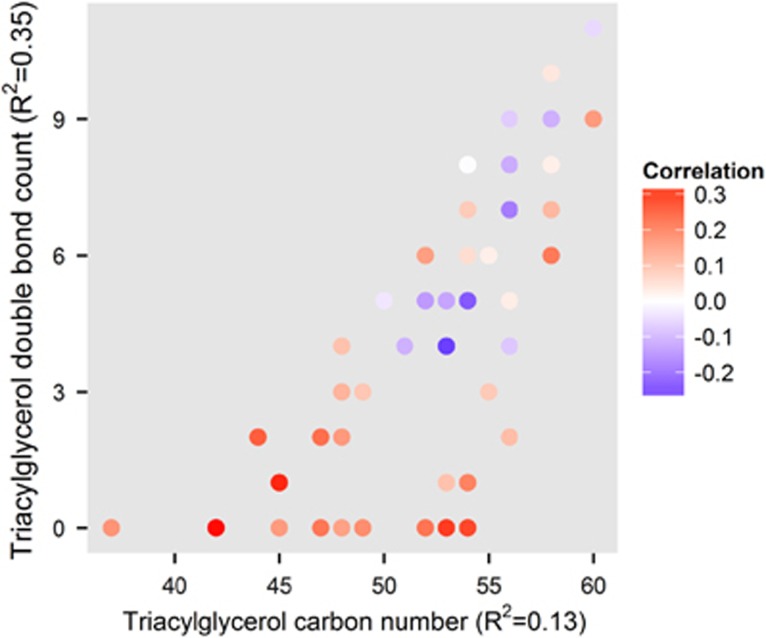
Association between the level of triacylglycerols (TGs) at baseline and the 2-month follow-up weight gain (Spearman correlation; color of the points) with respect to the number of carbon atoms (x-axis) and the number of double bonds between carbon atoms (*y* axis). The baseline levels of saturated and monounsaturated compounds (*y*=0 and *y*=1, respectively) are associated with short-term weight gain (red color). The coefficient of determination (*R*^2^) of the linear model for the association as a function of triacylglycerol carbon number and double-bond count are shown in the *x* axis and *y* axis labels, respectively (both *P*<0.05).

As it is well known that only specific TGs, those with low carbon number and double-bond count, associate with risk of type 2 diabetes, non-alcoholic fatty liver disease and insulin resistance,^28, 29, 30^ we set to examine how the TG composition associates with metabolic outcomes in FEP patients. Indeed, the baseline levels of TGs with a low double-bond count (including saturated and monounsaturated TGs) and a low carbon number were all positively associated with a 2-month change in the body mass index (Figure 3).

## Discussion

Our findings based on comprehensive metabolomics analysis in a prospective study setting demonstrate the potential of serum metabolites to predict metabolic abnormalities in FEP patients.

Our study corroborates several earlier metabolomics findings with respect to comparing psychotic patients and healthy controls as well as to examinations of pharmacometabolomic profiles of specific antipsychotic drugs. The strongest differences between the FEP cases and controls, both at baseline and at the 2-month follow-up, were observed for elevated polyunsaturated TGs and for the TG(54:2). The latter has been proposed earlier as a biomarker of schizophrenia.^13^ Increased levels of TGs in psychotic patients are well-documented.^42^ The observed deficiency of essential fatty acids has been previously attributed to drug-naive FEP.^43^

Abnormalities in sphingolipid metabolism have been previously reported in the early stages of schizophrenia^44, 45^ and are suggestive of disrupted myelin.^46^ In the present study, the baseline levels of sphingomyelin and PC clusters (LC3 and LC2, respectively) were associated with the 2-month improvement in the Brief Psychiatric Rating (BPRS). This indicates that aberrations in the levels of sphingolipids, which are important for the structure and function of cell membranes in the brain,^47^ might have potential for predicting the severity of the psychotic condition.

Similar differential effect of olanzapine and risperidone on the levels of PCs and TGs, as observed in the present study, has been reported previously.^12^ Also, the levels of oleic and palmitic acids have been reported to be altered by risperidone treatment,^48^ which is consistent with our observation that the metabolite cluster MC11 was downregulated.

PCs have earlier been associated with insulin responsiveness.^49^ In this study, the baseline levels of a PC clusters were found associated with 2-month increase in the insulin levels and waist-to-height ratio. The phosphatidylcholine cluster LC12 had a group effect, olanzapine effect as well as an association to follow-up changes in insulin level and waist-to-height ratio and global assessment of functioning, indicating a sensitivity to all three: the psychosis, its treatment and the development of metabolic syndrome.

LPCs have been considered as early biomarkers for the development of type 2 diabetes.^50^ In this study, the baseline levels of the LPC cluster (LC11) also were inversely associated with the 2-month change in the insulin level among lean subjects, indicating their potential as early predictors of metabolic syndrome.

Our study also suggests that weight gain in FEP patients is specifically associated with TGs with low carbon number and double-bond count, and not with TGs in general. These specific TGs are produced *de novo* in the liver^28, 51^ and their circulating levels also reflect the amount of liver fat.^29^ To our knowledge, this finding is novel and potentially clinically important. It suggests that patients with increased liver fat are at the highest risk of gaining weight as the psychosis develops and when the pharmacological treatment is initiated. These subjects are also at the highest risk of developing metabolic co-morbidities associated with psychosis and might therefore benefit from an early anti-obesity treatment together with the administration of antipsychotics—a hypothesis that needs to be tested in the future. Administration of metformin has already been suggested as an option to counteract antipsychotic-induced weight gain.^52^

The main limitation of the present study is the relatively small sample size, which did not allow us to develop diagnostic models predictive of metabolic outcomes of psychotic patients. However, the reported associations were also present in bootstrap-resampled data, providing the evidence of robustness. Nevertheless, because of the limited sample size, the current results should be seen as hypothesis generating and they need to be replicated in a larger study sample.

As the time series structure of the data is limited to the case group, it was not possible to fully untangle the temporal effects of the psychotic condition and the antipsychotic medication from the normal temporal variation over the follow-up. However, it is known that in healthy subjects, the metabolite levels are stable over time periods comparable to the follow-up time of this study.^53, 54^ The short period of antipsychotic treatment, already at the baseline sampling point for most subjects, sets another limitation to the full statistical separation of the effects of psychotic condition from the effects of antipsychotic medication. Further improvement in this part of the study design is hard to achieve due to the severity of psychotic condition but could result in a more precise characterization of aberrations that are directly related to psychosis. On the other hand, other studies have focused on short time periods similar to the first follow-up time point of the present study, whereas the last follow-up time point here is relatively far ahead, presenting new data on the medium-term effects. Even longer follow-up durations than in the present study could be useful for confirming the disease outcomes such as the eventual diagnosis of type 2 diabetes.

Taken together, the detected associations indicate that metabolite profiles may be used to identify those psychotic patients, who are the most vulnerable to developing metabolic co-morbidities. Future studies with larger sample sizes over longer periods of follow-up are needed for deriving and validating diagnostic models for the identification of subjects at highest risk of metabolic co-morbidities and for the prediction of their metabolic outcomes. Further, designed intervention studies are needed for assessing the preventive effects of an early anti-obesity treatment in this metabolic high-risk subgroup of FEP patients.

## Figures and Tables

**Table 1:** Baseline sociodemographic and clinical characteristics of the sample including FEP patients (*n*=36) and controls (*n*=19)

	*Control*	*Case, baseline*	*Case, 2 months*	*Case, 1 year*
Age	27 (23.7, 33.8)	24.5 (22, 29.5)	23.4 (21.7, 29.7)	26.7 (22.6, 33.4)
Brief Psychiatric Rating	24 (24, 25)	46 (39.5, 55)	36.5 (31.8, 43.2)	30.5 (25, 40.8)
Global assessment of functioning	90 (85, 90)	32 (30, 37.2)	38 (34.8, 40)	40 (37.2, 52.5)
Height	174 (168, 179)	175 (167, 183)	173 (166, 184)	172 (164, 181)
Male	10/19 (53%)	20/36 (56%)	11/24 (46%)	4/12 (33%)
Plasma glucose	4.2 (3.85, 4.42)	4.16 (3.84, 4.58)	4.2 (3.99, 4.41)	4.14 (4.06, 4.24)
Olazapine	—	12/36 (33%)	5/24 (21%)	3/12 (25%)
Risperidone	—	10/36 (28%)	5/24 (21%)	3/12 (25%)
Serum C-peptide	458 (367, 596)	553 (434, 868)	570 (433, 722)	544 (315, 720)
Serum HDL cholesterol	1.38 (1.34, 1.64)	1.37 (1.13, 1.54)	1.29 (1.15, 1.61)	1.22 (1.11, 1.67)
Serum LDL cholesterol	2.5 (2.11, 3.22)	2.86 (2.4, 3.5)	2.95 (2.43, 3.04)	2.64 (2.26, 3.3)
Serum insulin	7 (4.4, 9.35)	8.55 (6.15, 13.2)	8.35 (7.1, 14.2)	11.3 (8.15, 13.8)
Serum lipoprotein A-I	1.46 (1.37, 1.76)	1.42 (1.25, 1.52)	1.38 (1.23, 1.64)	1.37 (1.23, 1.68)
Serum lipoprotein B	0.69 (0.565, 0.86)	0.825 (0.66, 0.96)	0.815 (0.715, 0.935)	0.8 (0.61, 0.89)
Serum total cholesterol	4.58 (3.92, 5.6)	4.59 (4.21, 5.55)	4.79 (4.27, 5.34)	4.81 (4.15, 5.28)
Serum triglycerides	0.85 (0.71, 1.1)	1.1 (0.765, 1.37)	1.23 (0.792, 1.72)	0.98 (0.685, 1.94)
Waist	81 (76.5, 91)	83 (79, 89.2)	88.5 (82, 96.5)	91 (86.5, 97)
Weight	72.9 (63.1, 79.6)	69.7 (62, 79.1)	76 (60, 84.3)	74 (68, 95.8)

Abbreviations: FEP, first-episode psychosis; HDL, high-density lipoprotein; LDL, low-density lipoprotein.

Data shown as *n* (%), or median (25%, 75% quartiles).

**Table 2:** Description of the inferred metabolomic clusters

*Index*	*Size*	*Enrich.*	*TG median CN, DBC*	*Strongest identified peaks*	*Group eff. (median,* P*)*	*Olanzapine effects (median,* P*)*	*Risperidone effects (median,* P*)*	*Clinical associations, time (*R*,* P*)*
LC1	156			ChoE(14:0), ChoE(16:0), LPC(20:5), TG(14:0/16:0/17:0)+TG(14:0/18:0/15:0)				
LC2	120	PC, PE		PC(38:6) (2), PC(18:0/22:6), PC(16:0/22:5), PC(32:0)				BPRS, 2 m (−0.53, 0.0081)
LC3	88	SM		ChoE(18:1), SM(d18:1/16:0), SM(d18:1/22:0), SM(d18:1/24:0)				BPRS, 2 m (−0.54, 0.0075)
LC4	70			TG(53:6)				
LC5	69							weight, 2 m (-0.43, 0.036); BMI, 2 m (−0.42, 0.041)
LC6	58	TG	58, 8	PC(16:0/20:5), TG(56:7) (2), TG(56:8) (2), TG(22:6/18:1/18:1)				
LC7	56	PC, PE		ChoE(18:2), PC(36:2), PC(36:1), PC(36:4)		case, 2 m (0.75, 0.033)		LIPOA, 1 y (−0.72, 0.016)
LC8	54	TG	51.5, 2	TG(16:0/18:1/18:1), TG(18:1/18:1/18:1), TG(14:0/18:1/18:1)+TG(16:0/16:1/18:1), TG(50:1)			case, 2 m (−1.6, 0.031)	
LC9	52	TG	53, 1	TG(16:0/18:0/18:1), TG(16:0/18:1/20:1)+TG(18:0/18:1/18:1), TG(16:0/16:0/16:0)+TG(14:0/16:0/18:0), TG(17:0/18:1/18:1)			case, 2 m −1.2, 0.022)	LIPOB, 1 y (−0.69, 0.023); TCHO, 1 y (−0.63, 0.044)
LC10	51	PC		PC(34:2), PC(34:1), PC(36:3) (1), PC(38:3)				
LC11	48	LPC, LPE		LPC(16:0), PC(18:1/20:4), LPC(18:0), LPC(18:1)				LIPOA, 2 m (−0.57, 0.0057); TCHOH, 2 m (−0.49, 0.02); TCHOL, 1 y (−0.77, 0.0074); TCHOL, 1 y (−0.76, 0.0086); LIPOB, 1 y (−0.7, 0.02)
LC12	45	PC		PC(16:0/20:4), ChoE(20:4), SM(d18:1/24:1), PC(38:4)		case, BL (0.66, 0.032)		HOMA-IR, 2 m (0.5, 0.016); GAF, 2 m (-0.44, 0.034); insulin, 2 m (0.44, 0.036)
LC13	36	TG	46, 2	TG(14:0/16:0/18:1), TG(48:2), TG(46:1), TG(14:0/16:0/16:0)+TG(16:0/18:0/12:0)			case, 2 m (−1.1, 0.016)	CPEP, 2 m (−0.41, 0.045); LIPOB, 1 y (−0.72, 0.017)
LC14	34	TG	54, 4	TG(16:0/18:2/18:1)+TG(16:1/18:1/18:1), TG(18:1/18:2/18:1), TG(18:1/16:1/18:2)+TG(18:2/18:2/16:0), TG(54:5)				
LC15	32	TG	55, 6	TG(56:6), TG(54:5) (2), TG(54:6), TG(56:7)	case, BL-control, BL (0.81, 0.011); case, 2 m-control, BL (0.67, 0.041)			
LC16	32			PC(32:1), PC(30:0), PE(36:1) (1), LPC(14:0)				
LC17	32	TG	52, 4	TG(50:3), TG(52:5), TG(16:0/16:1/18:3)+TG(16:0/14:0/20:4), TG(54:6)	case, 1 y-case, BL (0.73, 0.042)			
LC18	32			PC(34:0)				weight, 1 y (−0.86, 0.0033); BMI, 1 y (−0.77, 0.013)
LC19	22							
LC20	19			TG(52:5)	case, BL-control, BL (−0.14, 0.016)			
LC21	12			PE(36:6e)				
LC22	9			SM(d18:0/24:1), TG(48:4) (1), PE(38:0e), TG(46:3)			case, BL (−0.48, 0.0067)	insulin, 2 m (0.56, 0.0066); HOMA-IR, 2 m (0.54, 0.0085); glucose, 2 m (0.5, 0.013); glucose, 1 y (0.77, 0.0049)
LC23	9			PC(36:3e), PC(38:4e), PC(38:3e), PC(40:4e)				WHtR, 2 m (0.52, 0.012); TRIG, 2 m (0.48, 0.022); GAF, 1 y (−0.65, 0.026); CPEP, 1 y (0.62, 0.035); TRIG, 1 y (0.65, 0.037)
LC24	7	HexCer		HexCer(d18:1/24:0), PE(42:0)				Insulin, 2 m (0.42, 0.047)
LC25	4				Case, 2 m-case, BL (0.4, 0.021)			
MC1	150		Glucose, urea, d-fructose					
MC2	26	Phosphate derivative	Glyceric acid-3-phosphate, valine, leucine, proline					LIPOA, 1 y (−0.91, 0.00078); TCHOH, 1 y (−0.78, 0.011)
MC3	25	Amino acid	Pyroglutamic acid, tyrosine, tryptophan,, arabinitol					glucose, 1 y (−0.68, 0.025)
MC4	23	Amino acid	Alanine, serine, glycine					
MC5	19		Lactic acid, octanoic acid, citric acid, aspartic acid					LIPOB, 2 m (−0.5, 0.028); LIPOB, 1 y (−0.81, 0.0072); TCHO, 1 y (−0.67, 0.039)
MC6	19	Carboxylic acid	Linoleic acid, stearic acid, octadecadienoic acid, arachidonic acid					
MC7	17		Creatinine, phenylalanine, ethanolamine, arabinofuranose					
MC8	17	Carboxylic acids	Uridine					LIPOB, 2 m (−0.52, 0.02); LIPOB, 1 y (−0.67, 0.039)
MC9	15	Sugar derivative						
MC10	11	Sugar derivative	d-Glucose, glucopyranose,, 2-deoxy-erythro-pentonic acid					GAF, 2 m (0.69, 0.00068); TRIG, 2 m (−0.49, 0.029); GAF, 1 y (0.66, 0.033)
MC11	11	Carboxylic acid	Glycerol, palmitic acid, oleic acid, palmitelaidic acid			case, BL (-0.98, 0.02)		CPEP, 2 m (0.44, 0.046)
MC12	11	Sugar derivative	Gluconic acid, 2-oxodeoxyhexodiulose, sorbose					
MC13	7							
MC14	6	Sugar derivative						glucose, 2 m (−0.57, 0.0084); TCHOL, 2 m (0.51, 0.024); CPEP, 1 y (0.63, 0.044)
MC15	6		Linolenic acid, eicosenoic acid, pentadecanoic acid	case, BL-control, BL (−0.71, 0.0013)				LIPOB, 2 m (0.54, 0.015)

Abbreviations: BMI, body mass index; BPRS, Brief Psychiatric Rating Scale; CN, carbon number; CPEP; C-peptide; DBC, double-bond count; GAF, global assessment of functioning; LC, lipid cluster; LIPOA, lipoprotein A-I; LIPOB, lipoprotein B; LPC, lysophosphatidylcholines; LPE, lyso-phosphatidylethanolamines; m, months; MC, metabolite cluster; PC, phosphatidylcholines; PE, phosphatidylethanolamines; R, Spearman correlation coefficient; SM, sphingomyelins; TCHO, total cholesterol; TG, triacylglycerols; TRIG, triglycerides; WHtR, waist-to-height ratio; y, years.

The table lists names of the clusters (‘Index'), number of peaks assigned to the cluster (‘Size'), enriched compound groups in the cluster (‘Enrich.' binomial test with false discovery rate at 0.01), median number of carbon atoms (‘CN') and unsaturated carbon–carbon bonds (‘DBC') in the TG-enriched clusters, strongest identified peaks, major differences between sample groups (case vs control, temporal change in the case group), major effects of the olanzapine and risperidone treatments, and associations to follow-up changes in clinical variables.

## References

[bib1] Foley DL, Morley KI. Systematic review of early cardiometabolic outcomes of the first treated episode of psychosis. Arch Gen Psychiatry 2011; 68: 609–616. 21300937 10.1001/archgenpsychiatry.2011.2

[bib2] Henderson DC, Vincenzi B, Andrea NV, Ulloa M, Copeland PM. Pathophysiological mechanisms of increased cardiometabolic risk in people with schizophrenia and other severe mental illnesses. Lancet Psychiatry 2015; 2: 452–464. 26360288 10.1016/S2215-0366(15)00115-7

[bib3] Kirkpatrick B, Miller BJ, Garcia-Rizo C, Fernandez-Egea E, Bernardo M. Is abnormal glucose tolerance in antipsychotic-naive patients with nonaffective psychosis confounded by poor health habits? Schizophr Bull 2012; 38: 280–284. 20558532 10.1093/schbul/sbq058 PMC3283143

[bib4] Correll CU, Robinson DG, Schooler NR, Brunette MF, Mueser KT, Rosenheck RA et al. Cardiometabolic risk in patients with first-episode schizophrenia spectrum disorders: baseline results from the RAISE-ETP study. JAMA Psychiatry 2014; 71: 1350–1363. 25321337 10.1001/jamapsychiatry.2014.1314

[bib5] Perez-Iglesias R, Martinez-Garcia O, Pardo-Garcia G, Amado JA, Garcia-Unzueta MT, Tabares-Seisdedos R et al. Course of weight gain and metabolic abnormalities in first treated episode of psychosis: the first year is a critical period for development of cardiovascular risk factors. Int J Neuropsychopharmacol 2014; 17: 41–51. 24103107 10.1017/S1461145713001053

[bib6] Leucht S, Cipriani A, Spineli L, Mavridis D, Orey D, Richter F et al. Comparative efficacy and tolerability of 15 antipsychotic drugs in schizophrenia: a multiple-treatments meta-analysis. Lancet 2013; 382: 951–962. 23810019 10.1016/S0140-6736(13)60733-3

[bib7] Keinanen J, Mantere O, Kieseppa T, Mantyla T, Torniainen M, Lindgren M et al. Early insulin resistance predicts weight gain and waist circumference increase in first-episode psychosis—a one year follow-up study. Schizophr Res 2015; 169: 458–463. 26589392 10.1016/j.schres.2015.11.002

[bib8] Bak M, Fransen A, Janssen J, van Os J, Drukker M. Almost all antipsychotics result in weight gain: a meta-analysis. PloS one 2014; 9: e94112. 24763306 10.1371/journal.pone.0094112 PMC3998960

[bib9] Le Hellard S, Theisen FM, Haberhausen M, Raeder MB, Ferno J, Gebhardt S et al. Association between the insulin-induced gene 2 (INSIG2) and weight gain in a German sample of antipsychotic-treated schizophrenic patients: perturbation of SREBP-controlled lipogenesis in drug-related metabolic adverse effects? Mol Psychiatry 2009; 14: 308–317. 18195716 10.1038/sj.mp.4002133

[bib10] Vehof J, Risselada AJ, Al Hadithy AF, Burger H, Snieder H, Wilffert B et al. Association of genetic variants of the histamine H1 and muscarinic M3 receptors with BMI and HbA1c values in patients on antipsychotic medication. Psychopharmacology (Berl) 2011; 216: 257–265. 21336576 10.1007/s00213-011-2211-x PMC3121946

[bib11] Nurmi EL, Spilman SL, Whelan F, Scahill LL, Aman MG, McDougle CJ et al. Moderation of antipsychotic-induced weight gain by energy balance gene variants in the RUPP autism network risperidone studies. Transl Psychiatry 2013; 3: e274. 23799528 10.1038/tp.2013.26 PMC3693401

[bib12] Kaddurah-Daouk R, McEvoy J, Baillie RA, Lee D, Yao JK, Doraiswamy PM et al. Metabolomic mapping of atypical antipsychotic effects in schizophrenia. Mol Psychiatry 2007; 12: 934–945. 17440431 10.1038/sj.mp.4002000

[bib13] Oresic M, Tang J, Seppanen-Laakso T, Mattila I, Saarni SE, Saarni SI et al. Metabolome in schizophrenia and other psychotic disorders: a general population-based study. Genome Med 2011; 3: 19. 21429189 10.1186/gm233 PMC3092104

[bib14] Oresic M, Seppanen-Laakso T, Sun D, Tang J, Therman S, Viehman R et al. Phospholipids and insulin resistance in psychosis: a lipidomics study of twin pairs discordant for schizophrenia. Genome Med 2012; 4: 1. 22257447 10.1186/gm300 PMC3334549

[bib15] He Y, Yu Z, Giegling I, Xie L, Hartmann AM, Prehn C et al. Schizophrenia shows a unique metabolomics signature in plasma. Transl Psychiatry 2012; 2: e149. 22892715 10.1038/tp.2012.76 PMC3432190

[bib16] McEvoy J, Baillie RA, Zhu H, Buckley P, Keshavan MS, Nasrallah HA et al. Lipidomics reveals early metabolic changes in subjects with schizophrenia: effects of atypical antipsychotics. PLoS One 2013; 8: e68717. 23894336 10.1371/journal.pone.0068717 PMC3722141

[bib17] Paredes RM, Quinones M, Marballi K, Gao X, Valdez C, Ahuja SS et al. Metabolomic profiling of schizophrenia patients at risk for metabolic syndrome. Int J Neuropsychopharmacol 2014; 17: 1139–1148. 24565079 10.1017/S1461145714000157

[bib18] Quinones MP, Kaddurah-Daouk R. Metabolomics tools for identifying biomarkers for neuropsychiatric diseases. Neurobiol Dis 2009; 35: 165–176. 19303440 10.1016/j.nbd.2009.02.019

[bib19] Ali-Sisto T, Tolmunen T, Toffol E, Viinamaki H, Mantyselka P, Valkonen-Korhonen M et al. Purine metabolism is dysregulated in patients with major depressive disorder. Psychoneuroendocrinology 2016; 70: 25–32. 27153521 10.1016/j.psyneuen.2016.04.017

[bib20] Kaddurah-Daouk R, Yuan P, Boyle SH, Matson W, Wang Z, Zeng ZB et al. Cerebrospinal fluid metabolome in mood disorders-remission state has a unique metabolic profile. Sci Rep 2012; 2: 667. 22993692 10.1038/srep00667 PMC3446657

[bib21] Oresic M, Hyotylainen T, Herukka SK, Sysi-Aho M, Mattila I, Seppanan-Laakso T et al. Metabolome in progression to Alzheimer's disease. Transl Psychiatry 2011; 1: e57. 22832349 10.1038/tp.2011.55 PMC3309497

[bib22] Han X, Rozen S, Boyle SH, Hellegers C, Cheng H, Burke JR et al. Metabolomics in early Alzheimer's disease: identification of altered plasma sphingolipidome using shotgun lipidomics. PLoS One 2011; 6: e21643. 21779331 10.1371/journal.pone.0021643 PMC3136924

[bib23] Kaddurah-Daouk R, Rozen S, Matson W, Han X, Hulette CM, Burke JR et al. Metabolomic changes in autopsy-confirmed Alzheimer's disease. Alzheimers Dement 2011; 7: 309–317. 21075060 10.1016/j.jalz.2010.06.001 PMC3061205

[bib24] Trushina E, Dutta T, Persson XM, Mielke MM, Petersen RC. Identification of altered metabolic pathways in plasma and CSF in mild cognitive impairment and Alzheimer's disease using metabolomics. PLoS One 2013; 8: e63644. 23700429 10.1371/journal.pone.0063644 PMC3658985

[bib25] Ahmed SS, Santosh W, Kumar S, Christlet HT. Metabolic profiling of Parkinson's disease: evidence of biomarker from gene expression analysis and rapid neural network detection. J Biomed Sci 2009; 16: 63. 19594911 10.1186/1423-0127-16-63 PMC2720938

[bib26] Bogdanov M, Matson WR, Wang L, Matson T, Saunders-Pullman R, Bressman SS et al. Metabolomic profiling to develop blood biomarkers for Parkinson's disease. Brain 2008; 131(Pt 2): 389–396. 18222993 10.1093/brain/awm304

[bib27] Hatano T, Saiki S, Okuzumi A, Mohney RP, Hattori N. Identification of novel biomarkers for Parkinson's disease by metabolomic technologies. J Neurol Neurosurg Psychiatry 2016; 87: 295–301. 25795009 10.1136/jnnp-2014-309676

[bib28] Kotronen A, Velagapudi VR, Yetukuri L, Westerbacka J, Bergholm R, Ekroos K et al. Saturated fatty acids containing triacylglycerols are better markers of insulin resistance than total serum triacylglycerol concentrations. Diabetologia 2009; 52: 684–690. 19214471 10.1007/s00125-009-1282-2

[bib29] Oresic M, Hyotylainen T, Kotronen A, Gopalacharyulu P, Nygren H, Arola J et al. Prediction of non-alcoholic fatty-liver disease and liver fat content by serum molecular lipids. Diabetologia 2013; 56: 2266–2274. 23824212 10.1007/s00125-013-2981-2 PMC3764317

[bib30] Rhee EP, Cheng S, Larson MG, Walford GA, Lewis GD, McCabe E et al. Lipid profiling identifies a triacylglycerol signature of insulin resistance and improves diabetes prediction in humans. J Clin Invest 2011; 121: 1402–1411. 21403394 10.1172/JCI44442 PMC3069773

[bib31] Wang TJ, Larson MG, Vasan RS, Cheng S, Rhee EP, McCabe E et al. Metabolite profiles and the risk of developing diabetes. Nat Med 2011; 17: 448–453. 21423183 10.1038/nm.2307 PMC3126616

[bib32] Mantyla T, Mantere O, Raij TT, Kieseppa T, Laitinen H, Leiviska J et al. Altered activation of innate immunity associates with white matter volume and diffusion in first-episode psychosis. PLoS ONE 2015; 10: e0125112. 25970596 10.1371/journal.pone.0125112 PMC4430522

[bib33] Ventura J, Lukoff D, Nuechterlein KH, Liberman RP, Green MF, Shaner A. Psychiatric Rating Scale (BPRS), expanded version (4.0): scales, anchor points, and administration manual. Int J Methods Psychiatr Res 1993; 3: 227–243.

[bib34] Castillo S, Mattila I, Miettinen J, Oresic M, Hyotylainen T. Data analysis tool for comprehensive two-dimensional gas chromatography/time-of-flight mass spectrometry. Anal Chem 2011; 83: 3058–3067. 21434611 10.1021/ac103308x

[bib35] Nygren H, Seppanen-Laakso T, Castillo S, Hyotylainen T, Oresic M. Liquid chromatography-mass spectrometry (LC-MS)-based lipidomics for studies of body fluids and tissues. Methods Mol Biol 2011; 708: 247–257. 21207295 10.1007/978-1-61737-985-7_15

[bib36] Pluskal T, Castillo S, Villar-Briones A, Oresic M. MZmine 2: modular framework for processing, visualizing, and analyzing mass spectrometry-based molecular profile data. BMC Bioinformatics 2010; 11: 395. 20650010 10.1186/1471-2105-11-395 PMC2918584

[bib37] R Core TeamR: A Language and Environment for Statistical Computing. R Foundation for Statistical Computing: Vienna, Austria, 2015; 10: 223.

[bib38] Rasmussen CE. The infinite Gaussian mixture model. Advances in Neural Information Process Systems 12 2000 pp 554–560.

[bib39] Kurihara K, Welling M, Vlassis NA. Accelerated variational Dirichlet process mixtures. Advances in Neural Information Process Systems 19 2007 pp 761–768.

[bib40] Lahti L, Knuuttila JE, Kaski S. Global modeling of transcriptional responses in interaction networks. Bioinformatics 2010; 26: 2713–2720. 20813878 10.1093/bioinformatics/btq500

[bib41] Benjamini Y, Hochberg Y. Controlling the false discovery rate: a practical and powerful approach to multiple testing. J Royal Stat Soc B 1995; 57: 289–300.

[bib42] Yan H, Chen JD, Zheng XY. Potential mechanisms of atypical antipsychotic-induced hypertriglyceridemia. Psychopharmacology (Berl) 2013; 229: 1–7. 23832387 10.1007/s00213-013-3193-7

[bib43] Khan MM, Evans DR, Gunna V, Scheffer RE, Parikh VV, Mahadik SP. Reduced erythrocyte membrane essential fatty acids and increased lipid peroxides in schizophrenia at the never-medicated first-episode of psychosis and after years of treatment with antipsychotics. Schizophr Res 2002; 58: 1–10. 12363384 10.1016/s0920-9964(01)00334-6

[bib44] Narayan S, Head SR, Gilmartin TJ, Dean B, Thomas EA. Evidence for disruption of sphingolipid metabolism in schizophrenia. J Neurosci Res 2009; 87: 278–288. 18683247 10.1002/jnr.21822 PMC2606914

[bib45] Narayan S, Thomas EA. Sphingolipid abnormalities in psychiatric disorders: a missing link in pathology? Front Biosci (Landmark Ed) 2011; 16: 1797–1810. 21196265 10.2741/3822

[bib46] McClay JL, Vunck SA, Batman AM, Crowley JJ, Vann RE, Beardsley PM et al. Neurochemical Metabolomics Reveals Disruption to Sphingolipid Metabolism Following Chronic Haloperidol Administration. J Neuroimmune Pharmacol 2015; 10: 425–434. 25850894 10.1007/s11481-015-9605-1 PMC4546545

[bib47] Svennerholm L. The Gangliosides. J Lipid Res 1964; 5: 145–155. 14174000

[bib48] Xuan J, Pan G, Qiu Y, Yang L, Su M, Liu Y et al. Metabolomic profiling to identify potential serum biomarkers for schizophrenia and risperidone action. J Proteome Res 2011; 10: 5433–5443. 22007635 10.1021/pr2006796

[bib49] Clore JN, Harris PA, Li J, Azzam A, Gill R, Zuelzer W et al. Changes in phosphatidylcholine fatty acid composition are associated with altered skeletal muscle insulin responsiveness in normal man. Metabolism 2000; 49: 232–238. 10690951 10.1016/s0026-0495(00)91455-0

[bib50] Cobb J, Eckhart A, Perichon R, Wulff J, Mitchell M, Adam KP et al. A novel test for IGT utilizing metabolite markers of glucose tolerance. J Diabetes Sci Technol 2015; 9: 69–76. 25261439 10.1177/1932296814553622 PMC4495543

[bib51] Kotronen A, Seppänen-Laakso T, Westerbacka J, Kiviluoto T, Arola JT, Ruskeepää A-L et al. Hepatic SCD1 activity and diacylglycerol but not ceramide concentrations are increased in the non-alcoholic human fatty liver. Diabetes 2009; 58: 203–208. 18952834 10.2337/db08-1074 PMC2606873

[bib52] Mizuno Y, Suzuki T, Nakagawa A, Yoshida K, Mimura M, Fleischhacker WW et al. Pharmacological strategies to counteract antipsychotic-induced weight gain and metabolic adverse effects in schizophrenia: a systematic review and meta-analysis. Schizophr Bull 2014; 40: 1385–1403. 24636967 10.1093/schbul/sbu030 PMC4193713

[bib53] Nicholson G, Rantalainen M, Maher AD, Li JV, Malmodin D, Ahmadi KR et al. Human metabolic profiles are stably controlled by genetic and environmental variation. Mol Syst Biol 2011; 7: 525. 21878913 10.1038/msb.2011.57 PMC3202796

[bib54] Floegel A, Drogan D, Wang-Sattler R, Prehn C, Illig T, Adamski J et al. Reliability of serum metabolite concentrations over a 4-month period using a targeted metabolomic approach. PLoS One 2011; 6: e21103. 21698256 10.1371/journal.pone.0021103 PMC3115978

