# Supplementary material for: Serum metabolite profile associates with the development of metabolic co-morbidities in first-episode psychosis
Source: Transl Psychiatry. 2016 Nov 15;6(11):e951–. doi: 10.1038/tp.2016.222 (PMC5314133; doi:10.1038/tp.2016.222)
Supplement: Supplementary Information [file tp2016222x1.pdf]

Supplementary Material to

Serum metabolite profile associates with the development of metabolic co-morbidities in first-episode psychosis

Supplementary Table 1. Diagnoses and baseline antipsychotic medication of the patients. The right column shows the number of subjects in the group (N) and the group's proportion in the study sample (%).

**Characteristic**	N (%)
**DSM-IV diagnosis:**	
Schizophrenia	18 (50%)
Schizoaffective disorder	2 (5.6%)
Schizophreniform disorder	4 (11.1%)
Delusional disorder	1 (2.8%)
Psychotic disorder NOS	6 (16.7%)
Major depressive disorder with psychotic features	1 (2.8%)
Bipolar I disorder with psychotic features	3 (8.3%)
Substance-induced psychotic disorder	1 (2.8%)
**Antipsychotic medication:**[1]	
Olanzapine	12 (33.3%)
Risperidone	9 (25.0%)
Quetiapine	8 (22.2%)
Aripiprazole	2 (5.6%)
Ziprasidone	1 (2.8%)
Sertindole	1 (2.8%)
First-generation antipsychotic	3 (8.3%)
No antipsychotic medication[2]	4 (11.1%)[2]

[1] Note: a patient may have been using more than one antipsychotic

[2] Includes two antipsychotic-naïve patients and two patients who in the interview reported having stopped using the prescribed antipsychotic

Supplementary Table 2. Association between the duration of medication at baseline and the lipidomic levels at baseline. Cluster name (left), Spearman correlation coefficient with its 95 % bootstrap confidence interval in parentheses (middle), and the Spearman test p-value (right) are shown in the table.

**Cluster name**	**Spearman correlation coefficient (confidence interval)**	**p-value**
LC 15 (TG)	0.36 (0.02, 0.65)	0.04
LC 6 (TG)	0.35 (0.00, 0.65)	0.04
LC 17 (TG)	0.33 (-0.02, 0.65)	0.06
LC 25	-0.32 (-0.65, 0.07)	0.07
LC 13 (TG)	0.26 (-0.15, 0.59)	0.13
LC 18	0.24 (-0.12, 0.55)	0.17
LC 16	0.22 (-0.14, 0.58)	0.21
LC 24 (HexCer)	0.22 (-0.18, 0.55)	0.21
LC 12 (PC)	-0.21 (-0.58, 0.16)	0.23
LC 20	0.19 (-0.25, 0.52)	0.29
LC 8 (TG)	0.18 (-0.22, 0.55)	0.29
LC 2 (PC, PE)	0.17 (-0.18, 0.50)	0.32
LC 10 (PC)	0.16 (-0.23, 0.52)	0.37
LC 14 (TG)	0.14 (-0.21, 0.45)	0.44
LC 11 (LPC, LPE)	0.12 (-0.23, 0.48)	0.49
LC 9 (TG)	0.12 (-0.28, 0.51)	0.50
LC 3 (SM)	0.10 (-0.27, 0.45)	0.58
LC 4	0.10 (-0.25, 0.43)	0.58
LC 22	0.10 (-0.30, 0.46)	0.59
LC 19	0.08 (-0.31, 0.47)	0.66

***Supplementary Table 3*.** Association between the duration of medication at baseline and the lipidomic levels at baseline. Cluster name (left), Spearman correlation coefficient with its 95 % bootstrap confidence interval in parentheses (middle), and the Spearman test p-value (right) are shown in the table.

**Cluster name**	**Spearman correlation coefficient (confidence interval)**	**p-value**
MC 11 (Carboxylic acid)	0.36 (-0.02, 0.66)	0.05
MC 15	0.35 (-0.03, 0.64)	0.05
MC 6 (Carboxylic acid)	0.19 (-0.21, 0.53)	0.31
MC 5	0.16 (-0.25, 0.55)	0.37
MC 1	0.14 (-0.21, 0.47)	0.44
MC 14 (Sugar derivative)	-0.12 (-0.48, 0.28)	0.50
MC 2 (Phosphate derivative)	0.11 (0.21, 0.42)	0.56
MC 10 (Sugar derivative)	0.09 (-0.28, 0.46)	0.64
MC 4 (Amino acid)	0.08 (-0.31, 0.43)	0.65
MC 12 (Sugar derivative)	0.06 (-0.31, 0.43)	0.74
MC 8 (Phenol)	0.06 (-0.32, 0.40)	0.76
MC 3 (Amino acid)	-0.06 (-0.38, 0.29)	0.76
MC 13	0.05 (-0.32, 0.44)	0.80
MC 7	0.02 (-0.37, 0.39)	0.92
MC 9 (Sugar derivative)	-0.01 (-0.38, 0.36)	0.96

Supplementary Figure 1. Difference between the median levels of FEP patients in olanzapine users vs. other treatment groups in the three time points (columns), shown for the clinical variables and metabolomics clusters (rows). Strongest differences (coupled Mann-Whitney U test and a bootstrap test of difference with $p<0.05$) are highlighted with the character "O". Significant enrichment of a functional group in a cluster (FDR<0.01) is shown in parenthesis following the cluster name.

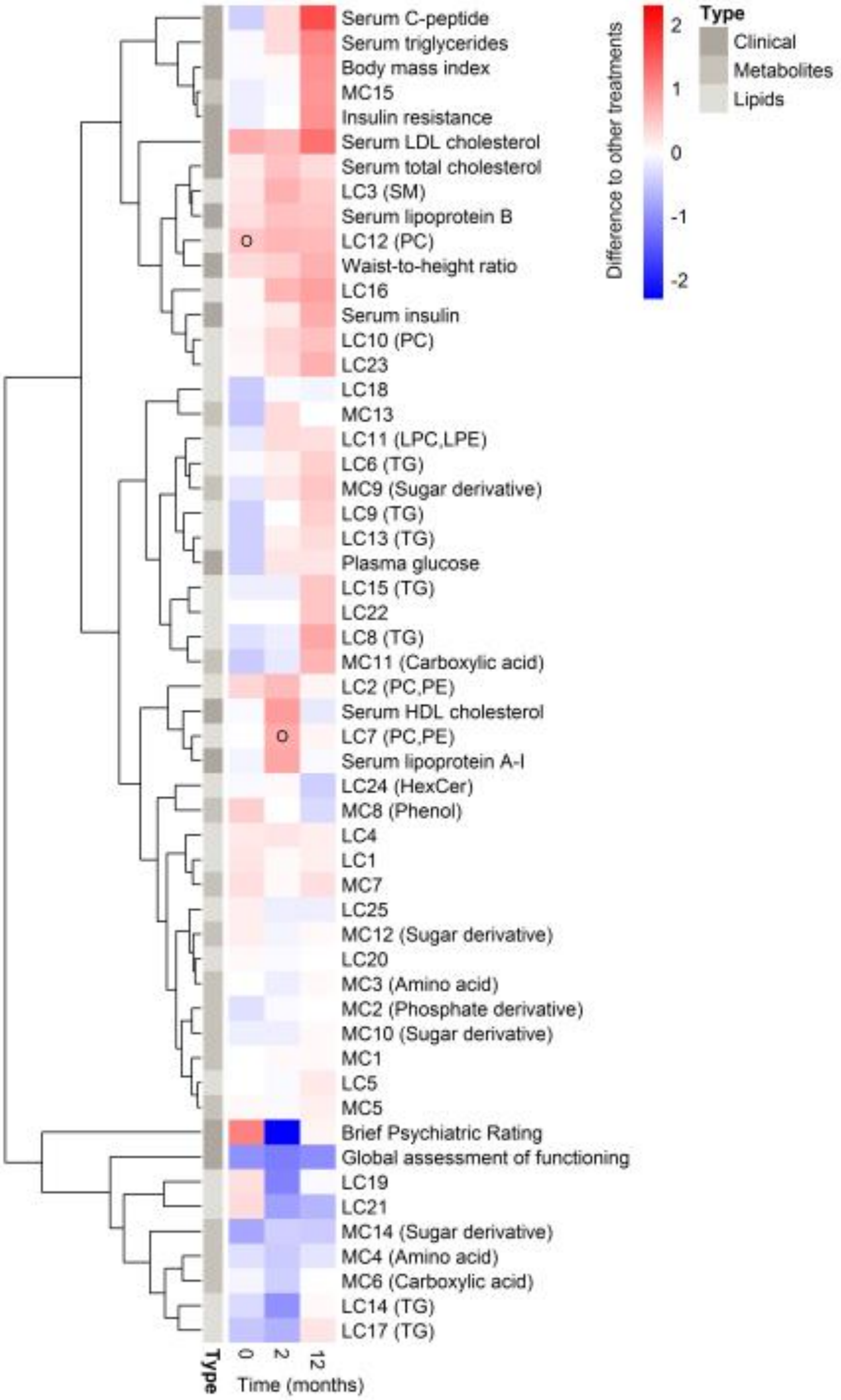

Supplementary Figure 2. Difference between the median levels of FEP patients in risperidone users vs. other treatment groups in the three time points (columns), shown for the clinical variables and metabolomics clusters (rows). Strongest differences (coupled Mann-Whitney U test and a bootstrap test of difference with p<0.05) are highlighted with the character "R". Significant enrichment of a functional group in a cluster (FDR<0.01) is shown in parenthesis following the cluster name.

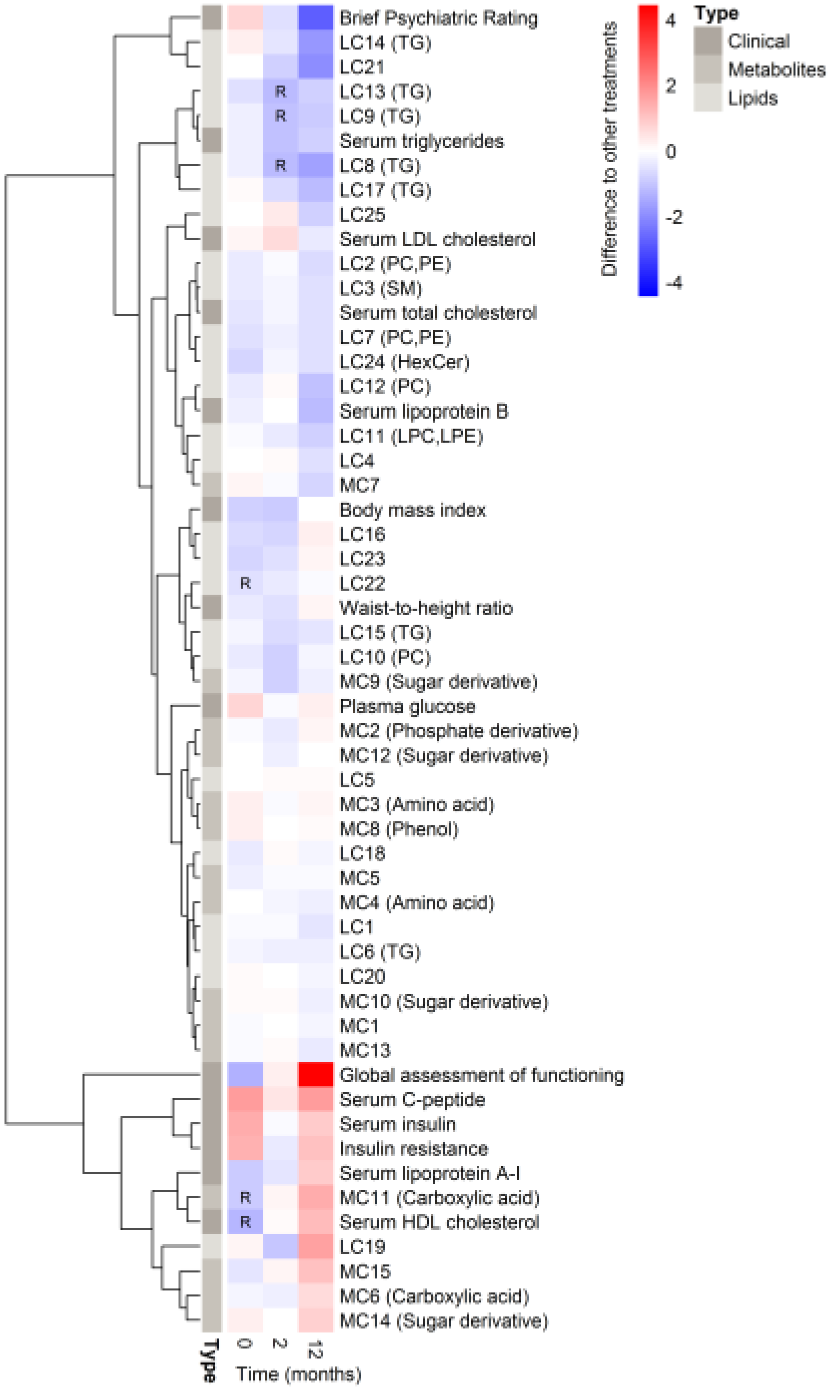

Supplementary Text 1

Supplementary information on lifestyle

The following questions on nutrition were asked from the participants:

(1) What kind of fat they usually use on bread
(2) What type of milk they usually drink (in terms of fat content)
(3) How often they eat full-fat cheeses
(4) How often they eat reduced-fat cheeses
(5) How often they fish
(6) How often they eat vegetables
(7) How often they eat fruits or berries
(8) How often they eat hamburgers, pizza or other savoury pastries
(9) How often they eat sweet pastries
(10) How often they eat chocolate or other sweets
(11) How often they drink sugary juices or soft drinks

We categorized question (1) as either vegetable oil-based or butter-based products. Their frequencies did not differ significantly between patients and controls (χ^2=0.01, P=0.94). Similarly, we categorized milk into milk with low (0-1%) or high fat content. This did not differ significantly either (χ^2=2.83, P=0.09).

The response categories for questions 2-11 were 0=no use, 1=on 1-2 days/week, 2=on 3-5 days/week and 3=on 6-7 days per week. We tested differences between cases and controls using the Mann-Whitney U test. There were no significant differences in any of the diet variables.

Physical activity was measured by Gothenburg scale (Wilhelmsen et al. 1972). The participants were classified as physically active if they reported exercising at least 3 hours per week. Patients were less likely to be physically active, but the difference did not quite reach statistical significance (χ^2=3.59, P=0.06).

Current smoking was more common in patients than in controls (37.9% vs. 10.5%, Fisher's exact test P=0.049).

The questions on nutrition and physical activity were taken from the Health behaviour among the Finnish adult population (Männistö et al. 2010).

References:

Männistö S, Laatikainen T, Helakorpi S, Valsta LM. Monitoring diet and diet-related chronic disease risk factors in Finland. Public Health Nutr. 2010 Jun;13(6A):907-14.

Wilhelmsen L, Tibblin G, Werko L. A primary preventive study of Gothenburg, Sweden. Prev Med 1972;1:153-60.
